# The Importance of Using Plant Resources During COVID-19 Pandemics to Mitigate Daily Needs and Some Diseases

**DOI:** 10.3389/fpubh.2022.823804

**Published:** 2022-03-25

**Authors:** Binsheng Luo, Arvind Bhatt

**Affiliations:** Lushan Botanical Garden, Jiangxi Province and Chinese Academy of Sciences, Lushan, China

**Keywords:** COVID-19, rural area, ethnobiology, food plant, medicinal plant

## Abstract

At the end of 2019, the coronavirus virus COVID-19 has brought the whole world a serious disaster. During this special time, some rural communities were least affected by the epidemic, mainly reflected on the rational utilization of natural biological resources, including edible and medicinal plants and the management of the home gardens. This paper deconstructed the self-responses of rural communities during the pandemic time and tried to provide some suggestions for local government on policymaking. In the end, the future development of ethnobiology in China has been discussed.

## Introduction

Since the end of 2019, COVID-19 has spread as a highly contagious pandemic to the world. Due to this, factories have stopped production, logistics has been almost interrupted, prices have soared, and home isolation measures have caused many cities to shut down. Although rural communities are rarely directly affected by the virus, they still suffer from shortages of medical supplies and food (1).

The famous ethnobiologist Pieroni and Price once mentioned: in many cultures, homemade plant-based beverages and foods are an important treatment method that communities rely on to treat minor, infectious, and chronic diseases (2). In 2020, a group of ethnobotanists studied the epidemic prevention responses in 17 indigenous communities worldwide and observed that many families were reusing the homemade botanical drugs or foods they used to apply for influenza and other respiratory symptoms (3). Among these cases, the most significant change in many areas was the increased consumption of ginger and garlic, followed by onions, turmeric, and lemons, which the locals believe to be good for treating the virus and boosting immunity (3). We have met similar cases in our ethnobotanical studies, during which the consumption of some plant resources has significantly increased. For example, we found (data unpublished) in the Baiku Yao community in Guangxi Province of China that many young people, who were supposed to work outside in cities were isolated in the villages because of the COVID-19, so they spontaneously went to the wild to collect firewood and wild food plants to meet their daily needs, like *Pistacia weinmanniifolia* and *Dioscorea persimilis*.

Thus, we assumed that the rural communities also have a certain mechanism of spontaneous reactions against disasters such as a daily supplies shortage caused by epidemic isolation, war, economic breaking up etc. This resistance ability must be based on rich biodiversity and well-preserved traditional knowledge of biological resource application. From the cases observed by Pieroni and us, these phenomena are worthy of study and consideration. Therefore, this paper intends to deconstruct the phenomena of using plant resources as a safety buffer to resist disasters in the rural community and discuss the role of ethnobiology and the potential role of traditional knowledge about natural recourses during a crisis like epidemic situation; simultaneously, put forward practical suggestions for local government decision-making reference.

## Local Health Line of Defense Against Sudden Disasters

### The Role of Home Gardens

When rural communities face disasters, the two most immediate problems are health issues and famine, usually reflected in utilizing edible and medicinal plants. The collection source usually includes the wild and home gardens.

As traditional agricultural systems, home gardens have highly diverse cultivated and domesticated plants used as food, spices, stimulants, medicines, beverages, fodder, etc., to satisfy the family's self-sufficiency and generate additional income (4). The rational management of the home gardens can provide the rural community with adequate energy and nutrients while facing sudden disasters, which effectively improves emergency survival (5). Accordingly, scientists have begun to focus on the ability of home gardens to help rural communities adapt to climate change-related challenges (6, 7). In summary, home gardens is not only a platform for rural households to sufficient daily needs but also an important tool to resist disasters.

### Collection and Utilization of Wild Plants

For the rural communities with less developed transportation, the locals are good at using the plant resources of the surrounding environment (8). Collecting food plants in the wild, especially starchy foods, can help local people survive famine and increase energy sources; for example, the collection and processing of *Caryota urens* powder by the Dulong people (9), the collection and consumption of *Amorphophallus konjac* and *Dioscorea subcalva* by Hani people (10). Wild vegetables and fruits can provide additional nutrition for humans, including different proteins, vitamins, trace elements, and adequate dietary fiber (11). For example, *Colocasia gigantea*, a common and important wild vegetable in southwest China, is reported to have a high dietary supplementary value (12). Compared with modern standardized cultivated vegetables, the collection and consumption of wild food plants are pollution-free and can provide more diversified dietary supplements, which are healthier than the daily diet in cities (13).

The consumption of dual-use plants is often incorporated into the daily eating habits of rural households to strengthen the body, treat chronic diseases, or other mild ailments (14). According to an ethnobotanical case in the Hakka community, local people like to use sun-dried edible medicinal plants for soup in their daily diet for “removing fire,” strengthening the body, dispelling wind, and dehumidification, like the use of *houttuynia cordata, Ricinus communis* stem, and *Thlaspi arvense* (15). We also noticed the increased use of some soup-making plants, which are believed locally to be positive for anti-virus and immunity-boosting during the epidemic (for example, stewed *crucian carp* soup with dried *houttuynia cordata*). Drinking herbal tea is common for daily health care in southern China. According to a report in southern China, a total of 238 species were recorded for the preparation of herbal tea, covering 27 related health diseases (16). The use of medicinal food plants has played a very positive role in promoting the health of rural communities.

Rural communities often have their own traditional medical care and collect wild medicinal plants for treatment for a long history. Modern science has recognized its positive role and incorporated a range of plant-derived drugs into modern drug therapy (17). For rural communities, using medicinal plants is still one of the most important health guarantees for local communities to resist disasters (17).

In addition to directly decocting, oral or external applying medicinal plants, the medicinal bath is also a typical way. Medicinal bath is common in southern China, and one of the typical cases is the report of Li et al. in Jinping Yao Autonomous County (18). The study documented 110 medicinal species used for the bath to treat various diseases such as rheumatism, skin diseases, fall injuries, and especially gynecological diseases (18). When having medicinal bath, contacting the medicine soup and inhaling the volatile components can directly stimulate the body to enhance immunity or treat diseases. In addition to medicinal plants, physical methods are also one of the important means of rural community health protection, including acupuncture, massage, stimulating acupoints to promote the immune system and physical health care.

### Spiritual Assistance

Spiritual comfort can bring stability and adjuvant therapy to rural areas. Positive emotions often bring positive feedback to the body and even better immunity. During the COVID-19 pandemic, in the famous Wuhan Fangcang Hospital, doctors actively encouraged patients to do tai chi and square dancing to ease their mood and relax, thus achieving a good effect adjuvant treatment (19). Religion and medical care are closely linked in many rural communities, such as the Bimo culture in the Yi ethnic communities (20). The daily practice of plant resources and culture has been reported to promote community unity and ease anxiety (3). Therefore, while assessing the private response of rural communities in the context of a pandemic, we should not only focus on the biological activities and pharmacological properties but also need to pay attention to their diverse cultural significance and importance.

## Suggestions

The rapid spread of COVID-19 has put tremendous pressure on the global Western medical system, especially in urban areas, when traditional medicinal knowledge has regained more attention, which fully addressed the importance of relevant knowledge and the urgency of being concerned and protected. Western health systems have been passive in tracking and treating cases in which people with underlying chronic health conditions are at great risk, highlighting not only the importance of disease prevention, health promotion and maintenance. In many cultures, traditional medical systems place great emphasis on active health measures rather than passive care. However, we still know little about the scientific basis of many traditional medical interventions, including the pharmacological activities of foods and drugs and the psychological effects of ritual practices on health and wellbeing, most of which have not been evaluated using modern laboratory techniques. Therefore, it is necessary to conduct ethnobiological investigations to record and scientifically evaluate traditional knowledge using modern techniques.

Because of globalization, the COVID-19 outbreak highlights the need to formulate preventive strategies to protect public health. Food and medicine are important tools for the community to maintain overall wellbeing in challenging times. We need to pay more attention to these foods and drugs used in community health systems not just to understand their biological activities; in particular, we need to design appropriate health strategies, raise broad awareness of their cultural importance, and observe the dynamic in actual use. In the context of COVID-19, local governments can selectively prepare for the cultivation and management of these resources plants to cope with the recurrence of similar disasters. For example, local governments can train community residents to prevent sudden disasters and guide them to optimize the planting structure in home gardens; a grain storage system can be established to provide diversified food supplements regularly. Additionally, local governments can also popularize traditional knowledge about the practice and protection of plant resources.

As a unit of human society, the community has specific psychological, cultural, and social relationships. When scientists and local governments conduct research and policy formulation, they should not only focus on the material basis and therapeutic effects, but also their spiritual cultures, such as understanding the social and cultural background and religious beliefs of community residents.

## The Ethnobiology Development in China

COVID-19's impact makes ethnobiology more like a “survival science,” which fully shows the importance of traditional plant knowledge and the urgent need to be protected and scientifically evaluated. Ethnobiologists in China should also be prepared to contribute to the investigation, evaluation, and dissemination of traditional health strategies. On this basis, the cooperation of scientists in different disciplines, including ethnobiology, biochemistry, pharmacology, psychology, immunology, etc., can open up new ways to enrich medical resources and change the paradigm from disease treatment to comprehensive nursing. In addition, ethnobiologist can serve as a bridge between local stakeholders and scientists and promote equitable access and benefit-sharing.

The traditional use of plants can be viewed from many angles, which are quite mysterious to the general public. There is an urgent need to find practical ways to gain wider public support for biodiversity conservation to be widely realized in practice. We should support rural community residents who use social media, self-media and other technologies to share information about traditional practices, especially those living in remote areas. The use of new media to let more people know the real rural community and traditional biological resources can better protect the practical knowledge and even bring additional benefits.

Compared with other classical disciplines, the development of ethnobiology in China is relatively weak and has received less attention. Therefore, the development of ethnic biology needs to keep pace with the times.

## Conclusion

The community-level reaction to supply shortage led by disasters rarely gets people's attention. During the epidemic of COVID-19, based on published papers and our observation, some rural communities showed a certain coping mechanism, which demonstrated the importance of traditional knowledge in the management and utilization of biological resources. Accordingly, local governments can also formulate more reasonable prevention strategies that integrate into local life. The COVID-19 pandemic also shows that it is very necessary for the development of ethnobiology in China. We hope that more people will join the ethnobiological research to create better wellbeing for rural communities and human society.

## Data Availability Statement

The original contributions presented in the study are included in the article, further inquiries can be directed to the corresponding authors.

## Author Contributions

BL: conceptualization and writing and revising the draft manuscript. AB: reviewed, revised the draft, and improved the language. Both authors contributed to the article and approved the submitted version.

## Funding

This work was supported by the Special Project of the Lushan Botanical Garden of the Chinese Academy of Sciences (2021ZWZX12).

## Conflict of Interest

The authors declare that the research was conducted in the absence of any commercial or financial relationships that could be construed as a potential conflict of interest.

## Publisher's Note

All claims expressed in this article are solely those of the authors and do not necessarily represent those of their affiliated organizations, or those of the publisher, the editors and the reviewers. Any product that may be evaluated in this article, or claim that may be made by its manufacturer, is not guaranteed or endorsed by the publisher.
